# Practical Hints on the Selection and Use of the Microscope

**Published:** 1877-10-20

**Authors:** 


Practical Hints on the selection and use of the Microscope, intended for beginners.—By John Phinn, editor *American Journal of Microscopy*: 2d edition, fully illustrated and enlarged. Industrial Publishing Co., N. Y. This is a valuable work, especially for beginners, and those not familiar with the use of the microscope.

